# Bactericidal Effect of Pterostilbene Alone and in Combination with Gentamicin against Human Pathogenic Bacteria

**DOI:** 10.3390/molecules22030463

**Published:** 2017-03-17

**Authors:** Wee Xian Lee, Dayang Fredalina Basri, Ahmad Rohi Ghazali

**Affiliations:** School of Diagnostic & Applied Health Sciences, Faculty of Health Sciences, Universiti Kebangsaan Malaysia, Kuala Lumpur 50300, Malaysia; lee.wx@mckl.edu.my (W.X.L.); rohi@ukm.edu.my (A.R.G.)

**Keywords:** pterostilbene, synergistic, time kill assay, antibiotic, bactericidal

## Abstract

The antibacterial activity of pterostilbene in combination with gentamicin against six strains of Gram-positive and Gram-negative bacteria were investigated. The minimum inhibitory concentration and minimum bactericidal concentration of pterostilbene were determined using microdilution technique whereas the synergistic antibacterial activities of pterostilbene in combination with gentamicin were assessed using checkerboard assay and time-kill kinetic study. Results of the present study showed that the combination effects of pterostilbene with gentamicin were synergistic (FIC index < 0.5) against three susceptible bacteria strains: *Staphylococcus aureus ATCC 25923*, *Escherichia coli O157* and *Pseudomonas aeruginosa 15442*. However, the time-kill study showed that the interaction was indifference which did not significantly differ from the gentamicin treatment. Furthermore, time-kill study showed that the growth of the tested bacteria was completely attenuated with 2 to 8 h treatment with 0.5 × MIC of pterostilbene and gentamicin. The identified combinations could be of effective therapeutic value against bacterial infections. These findings have potential implications in delaying the development of bacterial resistance as the antibacterial effect was achieved with the lower concentrations of antibacterial agents.

## 1. Introduction

Antibacterial therapy has been a keystone of modern medicine practice for the treatment of several pathological diseases [1,2]. Unfortunately, bacteria have developed genetic modifications and mobilized molecular defense mechanisms that are able to protect them against antibiotics [1]. Due to the emergence of multidrug-resistant pathogens, it is now standard clinical practice to use two or more antibacterial drugs with different mechanisms of action in an attempt to expand the antimicrobial spectrum, to prevent the emergence of resistant organisms, to minimize side effects, and to obtain synergistic antimicrobial activity [3,4].

Natural products and their derivatives have been recognized for many years as a significant source of new leads in the development of new pharmaceutical agents [5]. Phytoalexins are low molecular weight antimicrobial compounds that are produced by plants as part of the plant defense system against a wide range of pathogens and herbivores. Over the past few decades, research and commercial interest in stilbene phytoalexins have escalated. Many of them have been subjected to intense investigations in the light of their potential biological activities and possible pharmacological applications [6,7].

*trans*-3,5-Dimethoxy-4-hydroxystilbene (pterostilbene) is a phytoalexin compound found primarily in *Pterocarpus marsupium* heartwood [8,9] and several foods and drinks, including blueberries and grapevines [10,11]. Pterostilbene, a structural analog of resveratrol, has shown higher bioavailability than resveratrol due to the presence of the methoxy groups, making it advantageous as a therapeutic agent. The useful effects of pterostilbene are well documented and multiple studies have suggested that pterostilbene may have numerous preventive and therapeutic properties in a vast range of human diseases, including cardiovascular diseases, diabetes and cancer, which are attributed to its pharmacological effects such as antioxidant [12], anti-inflammatory, and anticarcinogenic properties leading to improved normal cell function and inhibition of malignant cells [13,14]. Like resveratrol, pterostilbene was also shown to possess antifungal activity against various grapevine pathogens [15]. More recent studies reported that pterostilbene was 5 to 10 times more effective than resveratrol in inhibiting the germination of conidia of *Botrytis cinerea* and sporangia of *Plasmopara viticola* [16]. However, to the best of our knowledge, evaluation of the susceptibilities of several clinical bacterial isolates of different species to pterostilbene has not been reported previously. As a result, this study was undertaken to study the antibacterial potency of pterostilbene and its combination with the standard antibiotic gentamicin against a variety of Gram-positive and Gram-negative bacteria.

## 2. Results

### 2.1. Minimum Inhibitory Concentration (MIC) Values of Pterostilbene and Gentamicin against Gram-Positive Bacteria

The antibacterial property of pterostilbene was quantitatively assessed against both Gram-positive and Gram-negative bacteria by determining their minimum inhibitory concentration (MIC) values. In Table 1, it is shown that the MIC value of pterostilbene was 0.025 mg/mL, whereas gentamicin was effective in the range of 1.56–6.25 μg/mL against Gram-positive bacteria.

Pterostilbene was effective in inhibiting the growth of all tested Gram-negative bacteria, except *Acinetobacter baumannii* ATCC 19606, with MIC values in the 0.5–0.025 mg/mL range (Table 2). Out of the two tested Gram-positive bacteria, pterostilbene was the only capable of inhibiting *Staphylococcus aureus* ATCC 25923 at MIC value 0.025 mg/mL. On the other hand, the antibiotic gentamicin used as a standard antibiotic drug was more potent than the tested pterostilbene compound with the MIC values of 1.56 μg/mL, 6.25 μg/mL, 3.13 μg/mL, 6.25 μg/mL against *Staphylococcus aureus* ATCC 25923, *Bacillus cereus* ATCC, *Escherichia coli* O157 and *Pseudomonas aeruginosa* ATCC 15442, respectively (Table 1 and Table 2). The most susceptible strains towards pterostilbene were *Staphylococcus aureus* ATCC 25923 and *Escherichia coli* O157 (0.200 mg/mL), followed by *Pseudomonas aeruginosa* ATCC 15442 (MIC = 0.025 mg/mL).

### 2.2. The Minimum Bactericidal Concentration (MBC) Pterostilbene against Gram-Positive and Gram-Negative Bacteria

The minimum bactericidal concentration (MBC) assay results are summarized in Table 3; an antimicrobial agent is considered bactericidal if the MBC value is not more than fourfold higher than the MIC value [17]. The MBC value is the least concentration which has a colony count of less than 10 on the agar plate. From Table 3, the MBC value of pterostilbene was similar to its MIC value against *Staphylococcus aureus* ATCC 25923, which was 0.200 mg/mL, indicating bactericidal activity. However, the MBC value of pterostilbene showed a bacteriostatic mode of action towards the other two susceptible bacteria strains, with MBC values more than fourfold higher than their respective MIC values.

### 2.3. FICI Values and Interaction Effects of Pterostilbene and Gentamicin Combinations against Gram-Positive and Gram-Negative Bacteria

The outcome for the antibiotic combination studies between pterostilbene and standard antibiotic gentamicin, determined by calculation of FIC index values, are presented in Table 4. The combination of pterostilbene and gentamicin displayed FIC values of 0.125, 0.3185 and 0.25 against *Staphylococcus aureus* ATCC 25923, *Escherichia coli* O157 and *Pseudomonas aeruginosa* ATCC 15442, respectively, which indicated a synergistic interaction. It was noted that pterostilbene markedly reduced the MIC value of gentamicin by sixteen fold against *Staphylococcus aureus* ATCC 25923, followed by eightfold and fourfold, respectively, against *Pseudomonas aeruginosa* ATCC 15442 and *Escherichia coli* O157. Our results indicate that the interaction of pterostilbene potentiated the activity of gentamicin. In the present study, only interactions with synergistic effects were selected for time-kill analysis as it would provide descriptive (qualitative) information on the pharmacodynamics of antimicrobial agents [18].

### 2.4. Time Kill Kinetic of Pterostilbene and Gentamicin Combinations against Gram-Positive and Gram-Negative Bacteria

**Figure 1:**
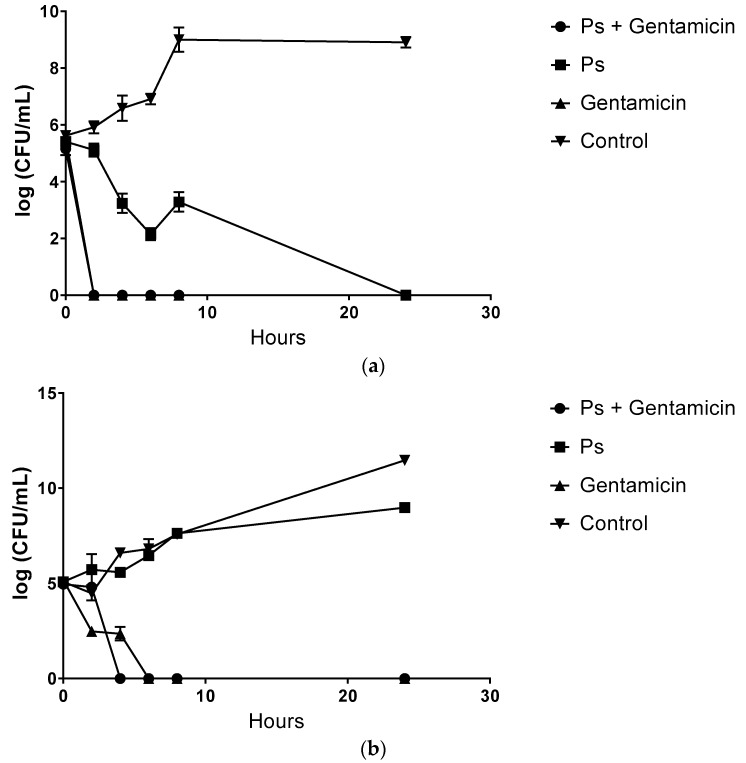

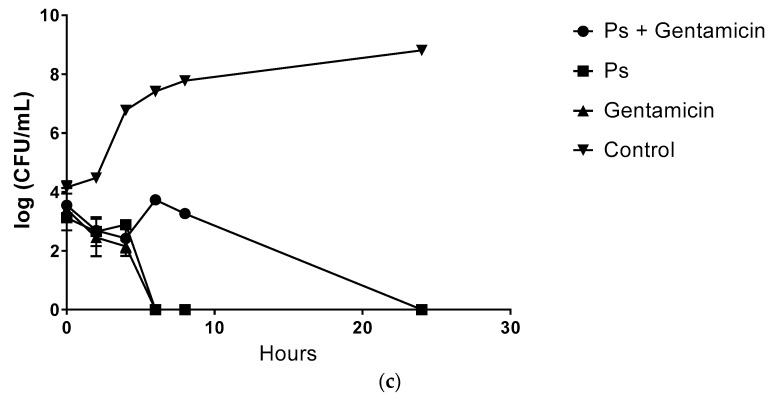
Time-kill growth curves of combination of pterostilbene with gentamicin, pterostilbene alone, and gentamicin alone against (**a**) *Staphylococcus aureus* ATCC 25923; (**b**) *Pseudomonas aeruginosa* ATCC 15442 and (**c**) *Escherichia coli* O157. The data is presented as a mean of 3 replicates.

Time-kill establishes the optimum time of exposure of pterostilbene against bacteria. The time-kill kinetic profiles of pterostilbene at 1 × MIC (Figure 1) displayed bactericidal activity towards *Staphylococcus aureus* ATCC 25923, showing a 3 log_10_ reduction in viable cell count relatively to the initial inoculum at 1 × MIC value after 5.5 h exposure. As expected from the determined MBC value, the time-kill analysis for pterostilbene against *Staphylococcus aureus* ATCC 25923 was consistent with the bactericidal characteristic. Kinetically, 1 × MIC of pterostilbene alone showed a slower rate of killing (5.5 h) compared to gentamicin alone as well as combination treatment (1.25 h).

On the other hand, combination treatment at ½ × MIC of both drugs had a similar killing rate compared to the most active agent gentamicin throughout the 24 h. However, the combination treatment showed indifference interaction with less than 2 log_10_ decrease in bacterial counts within a specific time period throughout 24 h between the combination and the most active agent. Figure 1b showed that pterostilbene at its MIC value did not demonstrate a reduction in bacterial count over time towards *Pseudomonas aeruginosa* ATCC 15442 relative to the initial inoculum. However, incubating the bacteria for 4 h resulted in an increase of the viable cell count ranging between 5.6 and 8.9 log_10_ CFU/mL. The time-kill assay curve in Figure 1b also indicates that the combination treatment showed an indifferent interaction between pterostilbene and gentamicin against *Pseudomonas aeruginosa* ATCC 15442. This is justified by the not more than 2 log_10_ increase in bacterial counts within a specific time period over 24 h between the combination and the most active agent. However, the combinations demonstrated more rapid killing after 3.8 h than gentamicin alone and the bacterial growth was completely attenuated after 4 h, whereas for gentamicin alone at the MIC complete bactericidal activity was exhibited after only 6 h. Our results suggest that pterostilbene combined with antibiotics might be microbiologically beneficial and synergistic. Our findings have potential implications in delaying the development of bacterial resistance as the antibacterial effect was achieved with lower concentrations of both drugs (antibiotics and stilbenes). In Figure 1c, the time-kill kinetics also showed a decrease in the number of surviving *Escherichia coli* O157 over the first 4 h, but the increase in bacterial counts from 2.59 and 3.72 log_10_ CFU/mL after 6 h indicated that after the initial bacteriostatic effect, regrowth of the organism could occur. Combination treatment failed to show a synergistic effect as indicated in time-kill curves showing an indifferent interaction through 24 h.

### 2.5. Scanning Electron Microscope (SEM) Analysis

**Figure 2:**
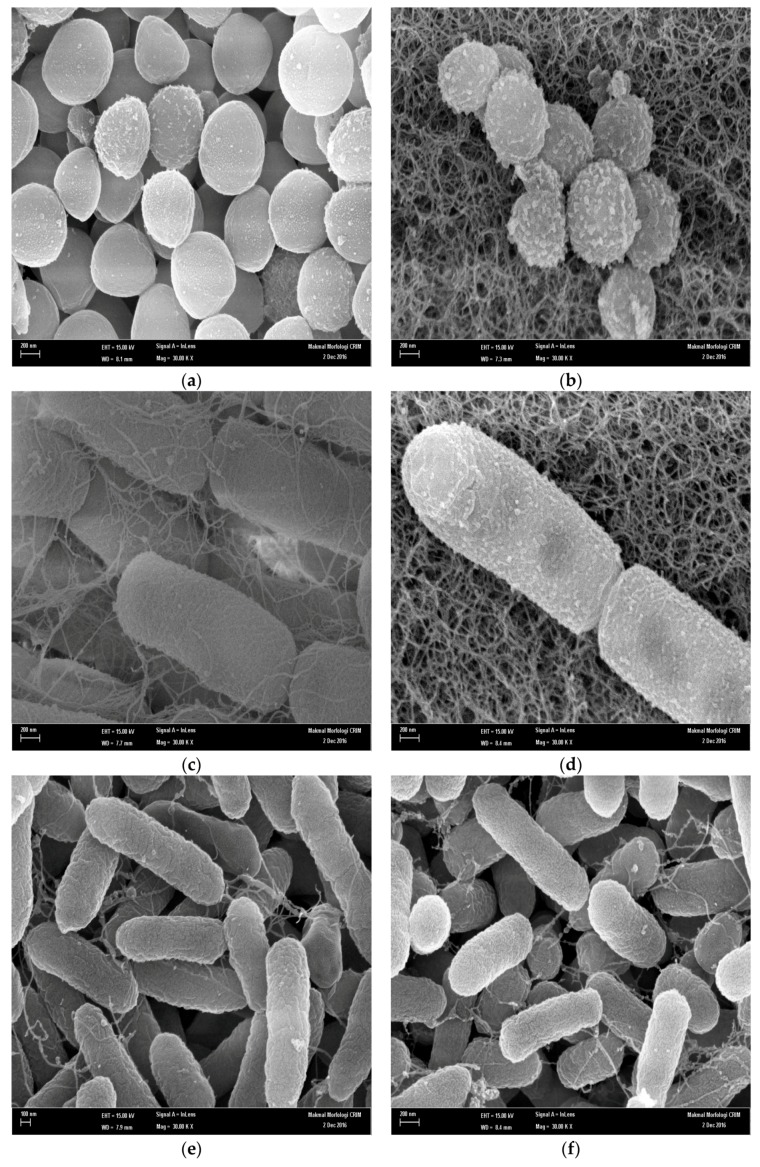
(**a**–**f**): Scanning electron microscope images of *Staphylococcus aureus* ATCC 25923 treated with pterostilbene at 1 × MIC (**a**) and control (**b**); *Escherichia coli* O157 treated with pterostilbene at 1 × MIC (**c**) and control (**d**) and *Pseudomonas aeruginosa* ATCC 15442 treated with pterostilbene at 1 × MIC (**e**) and control (**f**).

To investigate the interaction between pterostilbene and the bacterial cells, we decided to perform SEM analysis of bacterial cultures exposed to pterostilbene compound, in order to obtain additional evidence of the different effect observed in previous tests. As shown in Figure 2a,c, untreated *S. aureus* and *E. coli* exist as colonies of round-shaped and rod-shaped microbes, respectively, in the control culture. They have both smooth and intact cell walls. Morphological observation using SEM analysis of the *S. aureus* treated at 1 × MIC with pterostilbene showed that the quantity of *S. aureus* has greatly decreased and the bacteria became more irregular and unhomogenous in shape. A lot of blebs also appeared on the bacterial surface.

On the other hand, most of *E. coli* treated with pterostilbene at 1 × MIC maintained their rod shape but blebbing was notably visible on their surface. As for the *Pseudomonas aeruginosa* ATCC 15442, the mophologies of most of the survival cells remained unchanged, with rod-shapes and smooth surfaces (Figure 2e).

## 3. Discussion

**Figure 3:**
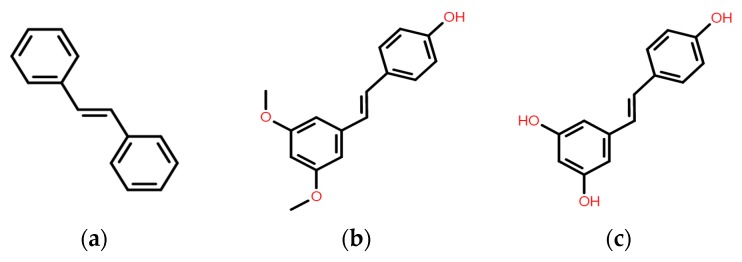
Chemical structures of (**a**) stilbene; (**b**) pterostilbene and (**c**) resveratrol.

Gram-positive and Gram-negative bacteria can cause serious infectious diseases in mammals, and the emergence of antimicrobial resistance has led to the evaluation of other agents with potential antimicrobial activity. For instance, gentamicin is an effective aminoglycoside when used against most bacterial infections, but its small therapeutic window, potential side effects and emergence of resistant strains limit its application [19]. Stilbenes are a new antimicrobial agent belonging to the polyphenol class which is primarily active against Gram-positive and negative pathogens [20]. Organic chemists have found *trans*-stilbenes, including resveratrol and pterostilbene (Figure 3), to be an intriguing structural class of compounds that may be considered ‘privileged structures’ because of their diverse array of biological and potential therapeutic properties [21,22]. As indicated by a huge number of published studies, resveratrol and its derivatives have yielded excellent potency and bactericidal activity. On the basis of the many promising reports on the activity of resveratrol and substituted stilbenes, the design of and synthesis of chemically novel analogs of these compounds was undertaken. Recent studies revealed that *E*-phenoxystyrenes and *E*-phenylthiostyrenes together with several substituted *E*-stilbenoid analogs were found to exhibit promising activity against Gram-positive bacteria [21]. An antimicrobial phenolic stilbene, (*E*)-3-hydroxy-5-methoxystilbene was recently isolated and shown to possess inhibitory activity against Gram positive bacteria, including isolates of methicillin-resistant *Staphylococcus aureus* (MRSA), *Mycobacterium bovis* BCG, and a virulent *Bacillus anthracis* (Sterne strain), among others. [23]. Pterostilbene is a resveratrol analogue with enhanced fungitoxic activity with respect to its precursor [24]. It was previously reported that the presence of a methoxy group in position 5′ together with a methoxy group in position 3′ confer high antimicrobial activity [20].

Preliminary study showed that pterostilbene possessed antibacterial activity against the reference strains of *Staphylococcus aureus* ATCC 25923, *Escherichia coli* O157 and *Pseudomonas aeruginosa* ATCC 15442. In comparison with gentamicin used as broad spectrum therapy against Gram-positive and Gram-negative infections, pterostilbene still showed 16 times lower antimicrobial activity against *Staphylococcus aureus* ATCC 25923 and *Escherichia coli* O157 and four times less inhibitory potency against *Pseudomonas aeruginosa* ATCC 15442. The same phenomenon was seen in the anti-MRSA effect of pterostilbene whereby both MRSA ATCC and clinical strains are less susceptible to pterostilbene compared to standard antibiotics [25]. On the other hand, the killing kinetics of *Pseudomonas aeruginosa* ATCC 15442 demonstrated bacteriostatic activity with less than 3 log_10_ CFU/mL reductions in colony count at 24 h compared with the starting inoculum. Consistent with the previous study, pterostilbene has been reported to exert bacteriostatic activity against MRSA strains with MBC values exceeding their MIC values [25]. In general terms, most of the phytochemicals or secondary metabolites from the plants are capable of inhibiting or slowing the growth of bacteria rather than killing the pathogen [23]. However, our study has showed that pterostilbene exert bactericidal effect on both *Staphylococcus aureus* ATCC 25923 and *Escherichia coli* O157. These results concur with the conclusions of bactericidal activity for pterostilbene from time-kill analysis, typically achieving 3 log_10_ CFU/mL kill over a period of 24 h.

The fundamental morphological differences in the cell wall and membrane organization of Gram negative and Gram positive organisms modulate their susceptibilities to phytoalexins in plants. Hence, Gram positive bacteria are often nevertheless susceptible to plant products. More importantly, studies also suggested that the combined effects of the mixture of natural compounds found in plants might be necessary to produce a synergistic antibacterial activity against Gram negative organisms [19]. However, pterostilbene was generally found active against Gram-negative bacteria with activity comparable with that against Gram-positive bacteria. Of course, several successful plant pathogens are nevertheless able to circumvent the toxic effects of these plant metabolites which can be observed from our study in both *Bacillus cereus* ATCC 11778 and *Acinetobacter baumannii* ATCC 19606, demonstrating no susceptibility to the pterostilbene compound. Combine antibiotic therapy may produce synergistic effects in the treatment of bacterial infection [26] as supported by previous study that phytochemical has the ability to delay the emergence of antimicrobial resistance [19]. Previous in vitro studies have reported synergistic combinations of various stilbene compounds, such as ε-viniferin and johorenol A against MRSA, both ATCC and clinical strains [3,4]. Synergistic antibacterial activity of two stilbene analogues (3,4,5-trihydroxystilbene and 3,5-dihydroxy-4-isopropylstilbene) purified from a *Bacillus* sp. N strain associated with entomopathogenic nematode *Rhabditis* (*Oscheius*) in combination with ciprofloxacin was also reported [20]. However, there was no literature concerning the role of pterostilbene in the antibacterial activity particularly the combination therapy.

In our experiments, the in vitro interactive effects of the antibiotics were determined by the broth microdilution checkerboard method as previously described. All combination treatment groups demonstrated synergism were subjected to retesting by time-kill kinetics studies. However, these results somewhat differed from the time-kill kinetics reports in which indifference bactericidal activity of pterostilbene against *Staphylococcus aureus* ATCC 25923 was observed. Comparison of the time-kill plots for the three organisms studied showed that the killing rate was the greatest for *Staphylococcus aureus* ATCC 25923, then *Escherichia coli* O157 and then with no bactericidal activity for *Pseudomonas aeruginosa* ATCC 15442. Likewise, gentamicin consistently showed bactericidal activity against *Staphylococcus aureus* ATCC 25923, then *Escherichia coli* O157 and *Pseudomonas aeruginosa* ATCC 1544. Considering the wide range of combination outcomes produced and the different killing rate from each compound, it is most likely that the antibacterial activity of pterostilbene and gentamicin is not attributable to one specific mechanism, but to several targets in the cell.

Damage to the structural integrity of the cells and considerable morphological alteration were confirmed by SEM analysis. The marked irregular shape of *Staphylococcus aureus* ATCC 25923 exposed to pterostilbene may have been the result of the extensive loss of cell contents, the exit of molecules and ions and cell lysis [22]. This was also supported by our results from the time kill assays demonstrating bactericidal action of pterostilbene against *Staphylococcus aureus*. It might be specific to the cocci bacteria, as no significant changes in cell shape were observed in *Escherichia coli* O157 which has remained its rod-shaped. In addition, the cell membrane surface in both treated *Staphylococcus aureus* ATCC 25923 and *Escherichia coli* O157 were rougher than that of the control, with pores and blebs appearing on the membrane surfaces. This considerable alteration in cell morphology was not observed in *Pseudomonas aeruginosa* ATCC 15442 treated with pterostilbene. This may be explained by the regrowth of the bacterial colonies after 4 h treatment as observed in time-kill analysis.

## 4. Materials and Methods

### 4.1. Preparation of Pterostilbene Compound

Purchased pterostilbene was in an amber bottle and each bottle contained 10 mg extracted pterostilbene (molecular weight: 256.3 g/mole). Pterostilbene (10 mg) was dissolved in 10% dimethyl sulfoxide (DMSO, 1 mL) to achieve the concentration of 10 mg/mL. The 10% DMSO solvent was prepared by adding 100% DMSO stock solution (0.1 mL) in distilled water (0.9 mL). The stock solution was kept in −4 °C till used.

### 4.2. Preparation of Bacterial Inoculum

The bacteria strains used in this study were cultured and maintained in Mueller Hinton agar (MHA) and incubated at 37 °C for 18 h. Isolated colonies were inoculated onto slant agar in a bijou bottle for storage at 37 °C for 24 h. The bacteria suspension was adjusted to a turbidity corresponding to a spectrophotometric absorbance at 0.08 at wavelength 650 nm, which is equivalent to 0.5 McFarland’s standard or a bacteria inoculum size of approximately 106 CFU/mL.

### 4.3. Determination of Minimal Inhibitory Concentration (MIC) and Minimum Bactericidal Concentration (MBC)

For the initial determination of the antibacterial activity of pterostilbene and gentamicin, a susceptibility screening study was conducted. The MIC values of pterostilbene and gentamicin against five bacterial strains were determined using the twofold serial microdilution method based on [27]. The tested pterostilbene and gentamicin were pippetted in a 96-well plate containing the sterile Mueller-Hinton broth enriched with 2% NaCl before the bacterial suspension at final inoculum of 106 CFU/mL was added. The range of final concentration of pterostilbene and gentamicin was 0.00078 mg/mL–0.2 mg/mL. A positive control comprised bacteria inoculum in Mueller-Hinton broth whereas the tested compounds in Mueller-Hinton broth were used as negative control to ensure medium sterility. The 96-well plate was then incubated at 37 °C for 24 h. MIC was the lowest concentration of compound and antibiotic showing no turbidity after 24 h, where the turbidity was interpreted as visible growth of bacteria. For consistency, each test was carried out in triplicate in a final volume of 0.1 mL per test well.

The MBC value was later determined by subculturing the well which showed no apparent bacterial growth on a sterile Mueller Hinton agar in an absence of antibacterial agent. The plate was incubated for 18 to 24 h. Bacterial growth on agar was observed and the concentration which has a colony count of less than 10 was considered as the MBC value. The MBC value was defined as the least concentration of antimicrobial agent that can kill >99% of the microorganism population where there is no visible growth on nutrient agar [28].

### 4.4. Determination of Fractional Inhibitory Concentration (FIC)

The combined antibiotic of pterostilbene with gentamicin against five strains of bacteria was evaluated from the FICI values for each combination using microdilution checkerboard technique [29]. The concentration of pterostilbene and gentamicin were prepared in five concentrations, namely 1/16 × MIC, 1/8 × MIC, 1/4 × MIC, 1/2 × MIC and 1 × MIC. Along the x-axis across the checkerboard plate, 50 µL of each pterostilbene was added into each well in the following sequence: 1/16 × MIC, 1/8 × MIC, 1/4 × MIC, 1/2 × MIC and 1 × MIC. As for the y-axis, 50 µL of vancomycin was added into each well in the same sequence as the tested pterostilbene. Inoculum size of approximately 106 CFU/mL was then added into all the wells. The well containing MHB and bacterial suspension served as positive control whereas well comprised only MHB and the tested antimicrobial agents served as negative control. The concentration of individual compound in the combination of pterostilbene and gentamicin which prevented visible bacterial growth was recorded as the MIC of the individual compound in the respective combination. The FICI value was then calculated as follows [29]:

FIC_index_ (∑ FIC) = FIC_A_ + FIC_B_ = A/MIC A + B/MIC B

where A = MIC of drug A in combination; B = MIC of drug B in combination; MIC_A_ = MIC of drug A alone; MIC_B_ = MIC of drug B alone Synergistic effect is defined as FICI of ≤0.5; partial synergism as 0.5 ˃ FICI ˂ 1; additivity as FICI = 1; indifference as 1 ˃ FICI ˂ 4; and antagonism as FICI of more than 4.

### 4.5. Time-Kill Study

Time-kill assays were performed by the broth macro-dilution method [29]. The rate of bacterial killing over time was performed only on the antibiotic combinations found to be synergistic by the microdilution checkerboard method. The time-kill kinetic study of the pterostilbene in combination with gentamicin against five strains of bacteria was performed in the microtiter 96-well plates. In each well, the combined agent was added to 0.04 mL Mueller-Hinton broth and 0.05 mL Inoculum suspension with approximately 10^6^ CFU/mL of exponentially growing cells were subsequently added. The growth control wells comprised only bacteria and 0.05 mL Mueller-Hinton broth. The wells were then incubated at 37 °C and viable counts were performed at 0, 2, 4, 6, 8, and 24 h after addition of antibacterial agents. At each hour, 0.01 mL of the sample was removed from the wells to be diluted twofold with normal saline (0.9% NaCl). The sample was then spread on Mueller-Hinton agar plates using cotton swab rod and incubated for 24 h at 37 °C. Colony count of bacteria of between 30 and 300 CFU/mL for each plate was determined to obtain time-mortality curves by plotting the log_10_ CFU/mL on the x-axis and time (h) on the y-axis. Synergistic interaction was defined as log_10_ decrease in CFU/mL between the combination and the most active agent at 24 h. Additive or indifference was defined as log10 CFU/mL reduction in colony count at 24 h by the combination compared with the most active single agent. Antagonism was described as log_10_ increase in CFU/mL after 24 h between the combination and the most active agent [30]. The time-kill curves were recorded as a decrease in CFU/m within a specific time period. Bactericidal and bacteriostatic were, respectively, defined as ≥3 log_10_ or <3 log_10_ reduction in colony count at 24 h compared with the starting inoculum [31].

### 4.6. Scanning Electron Microscopic (SEM) Analysis

In order to investigate the effect of pterostilbene on morphological changes of bacteria, treated and control cells were examined by scanning electron microscope (SEM) after 24 h treatment. The bacteria cells treated with 10% dimethyl sulfoxide (DMSO) were used as control. The cells were collected by centrifugation and washed with distilled water. The cells were fixed with 2% glutaraldehyde in 0.1 M phosphate buffer solution (PBS) and pH 7.4 for 15 min subsequently; the cells were washed three times and fixed with 1% osmium tetroxide in distilled water for 5 min at room temperature. The samples were dehydrated with a series of graded ethanol (70%, 90% and absolute ethanol), respectively for 5 min each, then coated with 42 nm thickness gold and examined on a Philips XL30 ESEM instrument (FEI Company, Hillsboro, OR, USA) at 28–30 kV [32].

## 5. Conclusions

The interaction between pterostilbene and gentamicin was bacteriostatic and enhancement of bactericidal activity of gentamicin against *Staphylococcus aureus* ATCC 25923, *Escherichia coli* O157 and *Pseudomonas aeruginosa* ATCC shown that these combinations have great potential worthy of further study and possible development as an alternative treatment in combating bacterial infections in the future.

## Figures and Tables

**Table 1:** Determination of MIC values of pterostilbene and gentamicin against Gram-positive bacteria.

Concentrations (mg/mL)	*Staphylococcus aureus* ATCC 25923		*Bacillus cereus ATCC 11778*	
	Pterostilbene	Gentamicin	Pterostilbene	Gentamicin
0.20000	−	−	+	−
0.10000	−	−	+	−
0.05000	−	−	+	−
0.02500	−	−	+	−
0.01250	+	−	+	−
0.00625	+	−	+	−
0.00313	+	−	+	+
0.00156	+	−	+	+
0.00078	+	+	+	+

+: presence of bacterial growth; −: absence of bacterial growth; Positive control comprises bacterial suspension and Mueller-Hinton broth; Negative control comprises Mueller-Hinton broth only.

**Table 2:** Determination of MIC values of pterostilbene and gentamicin against Gram-negative bacteria.

Concentrations (mg/mL)	*Escherichia coli* ATCC 35150		*Pseudomonas aeruginosa* ATCC 15442		*Acinetobacter baumannii* ATCC 19606	
	Pterostilbene	Gentamicin	Pterostilbene	Gentamicin	Pterostilbene	Gentamicin
0.20000	−	−	−	−	+	+
0.10000	−	−	−	−	+	+
0.05000	−	−	−	−	+	+
0.02500	+	−	−	−	+	+
0.01250	+	−	+	−	+	+
0.00625	+	−	+	−	+	+
0.00313	+	−	+	+	+	+
0.00156	+	+	+	+	+	+
0.00078	+	+	+	+	+	+

+: presence of bacterial growth; −: absence of bacterial growth; Positive control comprised bacterial suspension and Mueller-Hinton broth; Negative control comprised Mueller-Hinton broth only.

**Table 3:** Determination of MBC values of pterostilbene against Gram-positive and Gram-negative bacteria.

MIC (mg/mL)	Gram-Positive Bacteria	Gram-Negative Bacteria	
	*Staphylococcus aureus* ATCC 25923	*Escherichia coli* O157	*P. aeruginosa* ATCC 15442
0.20000	−	+	+
0.10000	−	ND	+
0.05000	−	ND	+
0.02500	−	ND	+
0.00125	ND	ND	ND

+: growth of bacteria on agar plate; −: no growth of bacteria on agar plate; ND: not done because the microtiter well at the tested concentration showed the presence of bacteria growth as shown in Table 1 and Table 2.

**Table 4:** Determination of FICI values and interaction effects of pterostilbene and gentamicin combinations against Gram-positive and Gram-negative bacteria.

Species	Antibacterial Agents	MIC (mg/mL)		FIC	
		Alone	Combination	FIC Individual	FIC Index
*Staphylococcus aureus* ATCC 25923	Pterostilbene	0.025	0.00157	0.0625	0.125 (S)
	Gentamicin	0.00157	0.0000981	0.0625	
*Escherichia coli* O157	Pterostilbene	0.2	0.0125	0.0625	0.3185 (S)
	Gentamicin	0.00313	0.0008	0.256	
*Pseudomonas aeruginosa* ATCC 15442	Pterostilbene	0.2	0.025	0.125	0.25 (S)
	Gentamicin	0.00625	0.00078	0.125	
